# Beyond the click: Pixel tracking technologies and patient data security in hospitals

**DOI:** 10.1093/pnasnexus/pgaf360

**Published:** 2025-12-09

**Authors:** Hilal Atasoy, Ryan McDonough, Guangyue (Maria) Zhang

**Affiliations:** Department of Accounting and Information Systems, Rutgers Business School, Rutgers University, New Brunswick, NJ 08854, USA; Department of Accounting and Information Systems, Rutgers Business School, Rutgers University, New Brunswick, NJ 08854, USA; Department of Accounting and Information Systems, Rutgers Business School, Rutgers University, New Brunswick, NJ 08854, USA

**Keywords:** online tracking technologies, pixel tracking, data privacy, data breaches, HIPAA compliance

## Abstract

Digital tracking technologies have transformed and enhanced online data collection across industries. Their integration into healthcare systems, however, raises urgent concerns about patient privacy and security. This study provides the first large-scale empirical analysis of pixel tracking technologies on US hospital websites and their unintended consequences for data breaches. Using historical website data from the Wayback Machine (2012–2023), we find that 66% of the sample employed pixel tracking, despite stringent privacy regulations. Our results reveal that third-party pixel use significantly increases data breach risk, underscoring a previously undocumented cybersecurity vulnerability. These findings highlight a critical regulatory gap in healthcare privacy, as tracking pixels operate outside the traditional scope of Health Insurance Portability and Accountability Act protections. As hospitals increasingly rely on digital tools, our study calls for reevaluating privacy and data security safeguards and regulatory oversight to address the emerging risks of modern tracking technologies.

Significance Statement Hospitals increasingly use tracking technologies, such as pixels embedded on their websites, to improve patient engagement and marketing. While common in other industries, these tools create unique privacy risks in healthcare because they share patient data with external parties without clear regulatory oversight. Analyzing hospital websites from 2012 to 2023, this study finds that using these tracking pixels significantly increases the chance of data breaches. These unintended disclosures expose sensitive patient information, undermining trust and highlighting gaps in current privacy protections. This research underscores the need for updated policies to better protect patients in a digital healthcare environment.

## Introduction

Digital tracking technologies have revolutionized online engagement, enabling organizations to collect user behavior data to enhance marketing and operational efficiencies. For instance, tracking pixels—small pieces of embedded code that transmit user data to external vendors—are widely used across industries. While broadly accepted in e-commerce and social media, their integration into the healthcare sector introduces unique ethical, security, and regulatory concerns. Yet, their implications for patient privacy and hospital cybersecurity remain largely unexplored.

Extant research has examined privacy regulations and data breaches in healthcare [e.g. (1–5)], as well as the security challenges associated with health information technologies and the digitalization of healthcare systems [e.g. (6–8)]. However, as hospitals increasingly integrate digital tracking tools, such as pixels, for web analytics, patient engagement, and marketing, these technologies introduce new and understudied risks. Unlike conventional electronic health record (EHR) systems or health information exchanges (HIE), tracking pixels operate beyond the boundaries of traditional IT security frameworks, transmitting user data to third-party vendors without direct hospital oversight.

Regulatory frameworks such as the Health Insurance Portability and Accountability Act (HIPAA) were established to safeguard patient data within traditional healthcare settings. These regulations were not designed to address the complexities of third-party digital tracking technologies, which collect, transmit, and potentially expose sensitive patient information to external entities. This creates a regulatory blind spot, where hospitals may inadvertently disclose protected health information (PHI) without explicit patient consent. The US Department of Health and Human Services (HHS) recently issued warnings about the risks of tracking pixels, signaling growing policy concerns. Recent reports suggest that many hospitals are sharing personal health information with third parties through pixel trackers installed on their websites (9–13). However, the consequences of such practices have not been previously quantified.

This study addresses this critical gap by comprehensively examining the historical adoption of pixel tracking technologies on US hospital websites. Leveraging data from the Wayback Machine, we construct a 12-year panel dataset (2012 to 2023) to track the evolution of pixel use over time. We further quantify the relationship between pixel tracking and hospital data breaches, providing the first systematic empirical assessment of whether digital tracking technologies compromise patient data security. Unlike prior cross-sectional studies that provide only static snapshots of pixel tracking [e.g. (9–11)], our longitudinal approach allows us to analyze trends and consequences of digital tracking in hospitals. This design enables us to control for unobserved time-invariant hospital characteristics and account for hospital-specific time trends to offer a causal assessment of how pixel tracking interacts with evolving data security risks. By capturing both temporal patterns and institutional variations, our study provides an empirical foundation for understanding the broader implications of digital tracking in healthcare.

The evidence we document collectively suggests that the heightened risks and vulnerabilities stem from the data-sharing practices with third-party tracking vendors. These results highlight that hospitals utilizing third-party pixels face significantly higher digital breach risks, underscoring the urgent need for updated privacy and security protections in the digital healthcare landscape. Our findings provide novel systematic evidence that a common marketing technology may inadvertently create cyber vulnerabilities in healthcare.

By bridging the fields of cybersecurity, health informatics, and regulatory policy, this study offers novel insights into an emerging digital threat. As hospitals increasingly adopt web-based technologies to improve patient engagement, the balance between innovation and data security becomes paramount. Our results contribute to ongoing policy discussions and call for stronger governance mechanisms or regulatory interventions to safeguard patient data in the era of pervasive digital tracking. Before presenting our findings, we outline the regulatory and technical context for pixel tracking in healthcare.

## Background: pixel tracking in healthcare

HIPAA was enacted in 1996 to protect patients' PHI, setting national standards for privacy and security in healthcare. The law applies to healthcare providers and their business associates, requiring safeguards to prevent unauthorized data access. The privacy rule mandates patient consent before PHI is disclosed, while the security rule enforces technical protections. However, these regulations were designed for traditional healthcare IT systems and do not explicitly account for third-party digital tracking technologies.

Recent concerns have emerged regarding tracking pixels, small embedded code snippets that collect user behavior data and transmit it to external vendors. Unlike cookies, which store data locally and can be deleted by users, pixels operate server-side, making them harder to detect and block. As third-party cookies are increasingly phased out due to privacy concerns, pixels have become a dominant tracking tool for measuring user engagement and targeting advertisements (14). Many hospitals use tracking pixels for marketing and website analytics, but these tools can inadvertently share PHI—such as IP addresses, appointment requests, and browsing behavior—with third-party platforms like Meta and Google.

Regulatory agencies have taken notice. The US Department of Health and Human Services (HHS) issued a bulletin in December 2022 clarifying that IP addresses linked to hospital webpages could be considered PHI, raising questions about HIPAA compliance (15). In 2023, HHS and the Federal Trade Commission (FTC) sent warning letters to 130 healthcare providers that their tracking pixels could compromise sensitive patient data (16). HIPAA mandates that hospitals remain responsible for breaches of PHI, regardless of whether the data loss occurs within their own IT systems or through third-party tracking tools. HHS has explicitly stated that hospitals transmitting PHI via pixels without safeguards may be subject to breach reporting requirements.

Real-world evidence confirms these risks: Community Health Network (CHN) disclosed in 2023 that a data breach affecting ∼1.5 million patients was directly linked to third-party tracking pixels transmitting PHI (17). In another case, Advocate Aurora Health reported a 3-million-patient breach in 2022, citing tracking pixels embedded on their website as the mechanism for unauthorized PHI disclosure to third parties such as Meta (18). Both hospitals reported these breaches under HIPAA, reinforcing that hospitals bear responsibility for data disclosures caused by tracking technologies.

Despite recent HHS guidance, legal challenges (e.g. a 2024 American Hospital Association lawsuit against HHS over its guidance) have created uncertainty about how HIPAA applies to pixel tracking. Beyond regulatory concerns, tracking pixels pose cybersecurity risks by increasing hospitals' exposure to third-party data breaches. Once patient data are transmitted to external vendors, hospitals have limited oversight of how it is stored or shared, making them vulnerable to security lapses in third-party systems. Cross-site tracking further heightens these risks, as third-party vendors can aggregate data from multiple websites, making it possible to reconstruct behavioral patterns and infer sensitive health details. Even when hospitals attempt to de-identify patient data, advanced tracking methods can re-identify individuals. These risks emphasize the need for stronger governance mechanisms to ensure that tracking technologies do not inadvertently expose patient data. As the debate over digital privacy intensifies, healthcare organizations must carefully weigh the benefits of online engagement against the potential risks to patient confidentiality and regulatory compliance. To empirically assess these risks, we next analyze hospitals' pixel use and its relationship with data breaches.

## Results

Results from Eq. 1, which include hospital and year fixed effects, are presented in Table 1. Each column introduces and progressively adds different control variables, as indicated in the table. Results indicate that pixel use increases data breach probability by at least 1.4% points (the smallest β1 coefficient = 0.014; standard error = 0.003). Given that the average breach probability in the sample is 3%, this represents a 46% relative increase, making it an economically meaningful effect.

**Table 1.** Pixel use and data breaches.

	Dependent variable: Breach*_i,t_*					
	(1)	(2)	(3)	(4)	(5)	(6)
Variable	Hospital FE	Size controls	System controls	Financial controls	IT security controls	Digitization controls
Pixel *t* – 1	0.014*** (0.003)	0.014*** (0.003)	0.014*** (0.003)	0.015*** (0.004)	0.016** (0.007)	0.016** (0.007)
Log number of employees		0.005 (0.005)	0.005 (0.005)	0.005 (0.005)	0.005 (0.004)	0.005 (0.004)
Log number of beds		−0.050 (0.045)	−0.051 (0.044)	−0.051 (0.045)	−0.043 (0.048)	−0.043 (0.048)
System size			0.002*** (0.001)	0.003** (0.001)	0.009* (0.005)	0.009* (0.005)
Log assets				0.001 (0.001)	−0.003 (0.003)	−0.003 (0.003)
Log net income				0.000 (0.000)	0.000 (0.000)	0.000 (0.000)
IT security system adoption					0.035 (0.036)	0.037 (0.036)
EHR adoption						0.001 (0.054)
HIE adoption						−0.007 (0.014)
Controls	No	Size	System	Financial	IT security	Digitization
Hospital fixed effects	Yes	Yes	Yes	Yes	Yes	Yes
Year fixed effects	Yes	Yes	Yes	Yes	Yes	Yes
Observations	10,324	10,324	10,324	8,535	3,195	3,195
*R* ^2^	0.414	0.415	0.417	0.419	0.545	0.545
Adj. *R*^2^	0.336	0.337	0.339	0.339	0.390	0.390

This table presents the main results from estimating Eq. (1). Refer to Table S1 for variable definitions. Standard errors are reported in parentheses and are clustered by hospital. ***P < 0.01. **P < 0.05. *P < 0.1.

To investigate the mechanisms linking pixel use to data breaches, we analyze breach types and pixel types separately.^a^ Data breaches occur for various reasons (see Table S4 for further details), but some are more plausibly related to pixel usage than others. We first focus on breaches categorized as unintended disclosure to third parties, as they are most directly related to pixel tracking. These breaches, reflecting unauthorized data sharing, follow a pattern consistent with our main findings: hospitals utilizing third-party tracking pixels experience a significantly higher likelihood of such breaches, corresponding to a 13% increase in unintended disclosures. This finding reinforces the concern that data transmission to external vendors via pixels introduces serious security vulnerabilities (Table 2, columns 1–4).

**Table 2.** Use different breach categories.

	Unintended disclosure Breach*_i,t_*				Physical Breach*_i,t_*			
Variable	(1)	(2)	(3)	(4)	(5)	(6)	(7)	(8)
Pixel *t* − 1	0.004**	0.004**	0.004**	0.005**	0.001	0.001	0.001	0.002
	(0.002)	(0.002)	(0.002)	(0.002)	(0.001)	(0.001)	(0.001)	(0.001)
Controls	No	Size	System	Financial	No	Size	System	Financial
Hospital FE	Yes	Yes	Yes	Yes	Yes	Yes	Yes	Yes
Year FE	Yes	Yes	Yes	Yes	Yes	Yes	Yes	Yes
Observations	10,324	10,324	10,324	8,535	10,324	10,324	10,324	8,535
*R* ^2^	0.285	0.287	0.287	0.399	0.154	0.154	0.154	0.154
Adj. *R*^2^	0.190	0.192	0.192	0.203	0.041	0.004	0.004	0.004

Refer to Table S1 for variable definitions. Standard errors are reported in parentheses and are clustered by hospital. ***P < 0.01. **P < 0.05. *P < 0.1.

Next, we examine breaches caused by physical losses (e.g. misplaced documents or devices), as these breaches are unlikely to be caused by pixel usage. In Table 2, columns 5–8, we analyze the relationship between pixels and physical breaches as a falsification test. Pixel trackers do not significantly affect breaches due to physical loss, aligning with theoretical expectations. Additionally, we further remove this category of physical losses from our analysis in Table 3, and confirm the robustness of our findings. These findings provide further confidence that the observed link between pixel tracking and breach risk is driven by digital vulnerabilities rather than unrelated security issues.

**Table 3.** Remove breaches due to physical document and device loss.

	Dependent variable: Breach*_i,t_*			
Variable	(1)	(2)	(3)	(4)
Pixel *t* − 1	0.012*** (0.003)	0.012*** (0.003)	0.012*** (0.003)	0.013*** (0.004)
Controls	No	Size	System	Financial
Hospital fixed effects	Yes	Yes	Yes	Yes
Year fixed effects	Yes	Yes	Yes	Yes
Observations	10,284	10,284	10,284	8,495
*R* ^2^	0.419	0.420	0.422	0.425
Adj. *R*^2^	0.341	0.342	0.344	0.345

Refer to Table S1 for variable definitions. Standard errors are reported in parentheses and are clustered by hospital. ***P < 0.01. **P < 0.05. *P < 0.1.

Our primary focus is on third-party pixels, which are used by 66% of hospital-years and pose a greater risk due to external data sharing. In contrast, 14% of hospital-years implement first-party pixels, which offer more control over data. First-party pixels allow hospitals to retain full control over collected data, providing tailored insights and mitigating risks linked to third-party sharing. In contrast, third-party pixels, often embedded as part of comprehensive analytics and marketing services offered by external vendors, facilitate cross-platform audience engagement and advanced advertising capabilities. As with many technology decisions, hospitals often outsource due to limited internal resources and expertise. Table 4 confirms that third-party pixels significantly increase data breach risks, reinforcing that external data sharing—not pixel technology itself—is the key vulnerability. First-party pixels, however, show no significant relationship with breaches, suggesting that external data transmission, rather than pixel technology itself, is the key risk factor.

**Table 4.** Third-party vs. own-pixel.

	Dependent variable: Breach*_i,t_*			
Variable	(1)	(2)	(3)	(4)
Third-party Pixel *t* − 1	0.011*** (0.003)	0.011*** (0.003)	0.011*** (0.003)	0.013*** (0.004)
Own Pixel *t* − 1	−0.004 (0.005)	−0.004 (0.005)	−0.004 (0.005)	−0.009 (0.011)
Controls	No	Size	System	Financial
Hospital fixed effects	Yes	Yes	Yes	Yes
Year fixed effects	Yes	Yes	Yes	Yes
Observations	10,205	10,205	10,205	8,416
*R* ^2^	0.245	0.246	0.246	0.248
Adj. *R*^2^	0.143	0.144	0.144	0.143

Refer to Table S1 for variable definitions. Standard errors are reported in parentheses and are clustered by hospital. ***P < 0.01. **P < 0.05. *P < 0.1.

These results are robust to several tests addressing potential endogeneity concerns, including reverse causality, selection bias, and unobserved confounding factors. Notably, our longitudinal fixed-effects design controls for time-invariant hospital characteristics our lead–lag analysis indicates that pixel adoption tends to precede breach incidents rather than result from them, supporting a causal link from tracking pixels to security risks. We also implement propensity score matching, hospital-specific time trends, and Heckman selection models to mitigate concerns about selection bias, parallel trends assumption, and nonrandom availability of archived websites. Nevertheless, as with any observational study, we acknowledge that we cannot fully rule out the influence of unobserved time-varying confounders or directly observe the causal chain between pixel use and specific breach events. It is also possible that hospitals adopting pixels may differ in unmeasured ways, such as in digital governance practices that correlate with breach risk. Despite these limitations, the consistency of our results across multiple sensitivity analyses strengthens our confidence that the observed relationship reflects a substantive link between third-party pixel tracking and increased breach risk. Full details of the threats to causal inferences and associated tests are provided in the Materials and methods section and Supplementary materials.

## Discussion

This study examines the intersection of technology and healthcare, analyzing the widespread adoption of pixel tracking technologies by US hospitals and their implications for patient privacy and data security. Our findings show that 66% of the sample observations employed tracking pixels between 2012 and 2023. The pervasive use of tracking pixels reflects hospitals' increasing reliance on digital tools for patient engagement and operational efficiency. Despite heightened regulatory scrutiny, hospitals continued to use tracking pixels through 2023, suggesting that voluntary curtailment has not occurred. Our analysis reveals that third-party pixel use significantly increases the risk of data breaches, whereas first-party pixels have no measurable impact on breach risk. These findings indicate that the security risks stem from data sharing with third-parties rather than from pixel technology itself.

Our findings highlight the trade-off between innovation and security in healthcare. While pixels enable hospitals to optimize resource allocation, improve website functionality, and better understand patient behavior, they also introduce vulnerabilities by transmitting sensitive data to third-party vendors. Unlike first-party pixels, which allow hospitals to retain control over collected data, third-party pixels expose patient information to external actors, often beyond hospital oversight. These risks are particularly concerning in the context of HIPAA regulations, as hospitals may inadvertently disclose protected health information (PHI) without explicit patient consent. The lack of direct control over third-party data practices further complicates compliance efforts, increasing hospitals' exposure to regulatory penalties and legal scrutiny.

Beyond compliance, pixel tracking raises broader ethical concerns about patient trust and transparency. The unauthorized transmission of PHI to external vendors may undermine public confidence in healthcare institutions, exacerbating existing concerns over data privacy in the digital age. As regulatory scrutiny intensifies, hospitals will likely face mounting pressure to reassess their digital tracking strategies.

Recent lawsuits against Meta reveal that third-party tracking vendors may mishandle or aggregate patient data received via hospital pixels. These cases illustrate a broader issue: hospitals may initially be unaware of the data-sharing risks posed by tracking pixels but are later required to report breaches once regulatory investigations expose unauthorized disclosures. Legal actions also suggest growing pressure on hospitals to reassess digital marketing tools, as failure to monitor third-party data practices can lead to regulatory penalties or class-action lawsuits.

Recent guidance from the US Department of Health and Human Services (HHS) underscores the urgency of this issue, signaling potential regulatory shifts that could impose stricter limitations on hospitals' use of tracking technologies. Additionally, the legal battle between the American Hospital Association and HHS highlights the ongoing tension between regulatory enforcement and hospitals' reliance on digital marketing tools to enhance patient outreach and service optimization.

Beyond regulatory reforms, hospitals can adopt institutional mechanisms to mitigate the risks posed by third-party pixel tracking. These include revising vendor contracts to explicitly restrict data sharing and require compliance with HIPAA-equivalent safeguards, implementing centralized oversight for web technologies within the hospital's compliance or IT security units, and increasing transparency by disclosing tracking practices in patient-facing privacy policies. Hospitals can also adopt opt-in consent mechanisms or shift toward first-party analytics solutions that retain data within the institution. These steps offer actionable measures for improving digital governance, even in the absence of immediate regulatory changes.

Although our study focuses on the healthcare sector, its implications extend to other industries handling sensitive data, such as finance and insurance, government services, and education [e.g. (19, 20)]. As digital tracking technologies become more pervasive across the board [e.g. (21)], the challenge of balancing technological innovation with robust privacy protections will only grow more pressing. Our findings emphasize the need for industry-wide governance frameworks that mitigate privacy risks while allowing organizations to leverage digital tools responsibly.

In conclusion, this study provides empirical evidence on the widespread use of pixel tracking by US hospitals and its associated security risks. While pixel technologies offer valuable benefits, they also introduce substantial vulnerabilities that hospitals must address. To safeguard patient privacy, healthcare institutions must critically evaluate their use of tracking pixels, strengthen data governance policies, and ensure compliance with evolving regulatory standards. As digital healthcare continues to expand, policymakers and hospital administrators must take proactive steps to balance technological advancement with the fundamental need for patient data protection, such as updating regulatory guidance to explicitly cover third-party tracking or requiring greater transparency and consent for data sharing via pixels. In an era of pervasive digital tracking, safeguarding patient data is not just a regulatory requirement but essential to maintaining trust in healthcare.

## Materials and methods

### Data sources

We examine pixel tracking adoption in large US hospitals, which tend to have complex digital infrastructures and higher online patient engagement, making them more likely to employ web-based tracking tools. Using the Centers for Medicare and Medicaid Services (CMS) Provider of Services File and Newsweek's 2023 World's Best Hospital list, we construct a panel of 1,201 unique hospitals from 2012 to 2023. To track pixel usage, we retrieve archived hospital websites via the Wayback Machine and analyze them using webXray, a tool that detects third-party tracking technologies.

Our final dataset consists of 11,013 hospital-year observations, after excluding cases where archived websites were unavailable. We supplement this with CMS data to capture hospital characteristics such as size, financials, ownership structure, and teaching status. Additionally, we incorporate HIMSS data (2012–2017) to measure electronic health record (EHR) adoption (22), IT security system implementation, and health information exchange (HIE) participation, which serve as proxies for hospitals' digitalization level and data governance capabilities.^b^

Detailed descriptions and sources of all variables used are presented in Table S1, and descriptive statistics are presented in Table S2.

### Identification of pixel tracking technologies

We detect tracking pixels using webXray (23), which identifies third-party requests on hospital websites, including invisible image-based pixels and network file types (fetch, xhr, beacon, or script) (Detailed steps are presented in Fig. S1).^c^ To ensure accuracy, we exclude first-party requests linked to hospital system-owned domains. Our longitudinal approach (2012–2023) allows us to examine trends in pixel adoption over time, addressing prior studies' limitations of focusing only on single points in time [e.g. (24–27)]. Cross-sectional analyses provide only static snapshots of tracking technologies during page loading tracking, whereas, a panel dataset enables us to track the evolution and consequences of pixel usage in hospitals. This approach is particularly important given the shifting regulatory landscape and growing awareness of digital privacy risks. By leveraging panel data, we can control for time-invariant, unobserved hospital characteristics, thus mitigating concerns about omitted variable bias. Additionally, incorporating hospital-specific time trends allows us to account for broader technological advancements and policy changes that may influence both pixel adoption and breach risk.

**Fig. 1.**
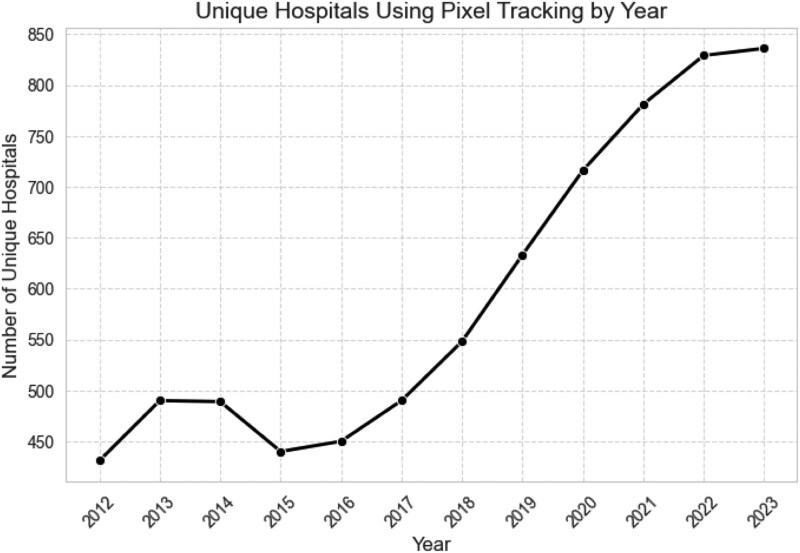
Pixel use over time.

We find that pixel adoption exhibits a general upward trend, highlighting hospitals' continued reliance on tracking technologies for patient engagement and marketing (Fig. 1; detailed yearly statistics are presented in Table S3). Despite rising privacy concerns and regulatory scrutiny, we find no substantial decline in pixel usage in recent years.

### Data breaches

We identify hospital data breaches using the Privacy Rights Clearinghouse Data Breach Chronology Database (28), which categorizes breaches by industry. Healthcare remains the most frequently breached sector, surpassing all others, including business and finance. In our sample, 3% of hospital-year observations report a data breach.

### Statistical analyses

To assess whether pixel use increases breach probability, we estimate the following hospital and year fixed-effects model:


(1) $$\mathrm{Breac}h_{it}=\alpha +\;\beta_{1}\mathrm{Pixel}\;\mathrm{Us}e_{it-1}\;+\sum \gamma_{k}\mathrm{Control}s_{it-1}\;+\;\eta \mathrm{Year}\;\mathrm{FE}+\varphi \mathrm{Hospital}\;\mathrm{FE}\;+\varepsilon_{it}$$

$\mathrm{Breac}h_{it}$ is an indicator variable reflecting whether hospital *i* had a breach incident in year *t*. Our main independent variable of interest is Pixel, which is an indicator variable that is equal to one if a hospital website has a pixel, and zero otherwise. Our main coefficient of interest is $\beta_{1}$, which estimates the effect of Pixel in the previous year on the breach probability in the current year. We utilize lagged values of Pixel to reduce the simultaneity concerns between the breach incidents and pixel use. We control for hospital-level characteristics, including Number of Beds, Number of Employees, System Size, Net Income, and Assets. Hospital fixed effects account for time-invariant unobserved heterogeneity across different healthcare organizations. Year fixed effects control for the time-specific shocks experienced by the healthcare market. Standard errors are clustered by hospital.

Estimating the relationship between pixel use and data breaches presents several identification challenges. First, selection bias may arise if hospitals that adopt tracking pixels systematically differ from those that do not. To address this, we apply propensity score matching to create a balanced comparison group, ensuring that hospitals with and without pixels are similar across key characteristics.

Second, time-varying differences in breach risk could violate parallel trends assumptions. We assess the temporal relationship between pixel adoption and breaches (29), finding that pixel adoption precedes breaches, rather than breaches prompting hospitals to adopt tracking technologies (Table S7). Additionally, we incorporate hospital-specific time trends to control for unobserved time-variant factors (Table S6).

Third, pixel adoption could proxy for weaker security practices, potentially confounding results. To mitigate this, we control for IT Security System Adoption, EHR Adoption, and HIE Adoption, which influence breach risk (7, 30–32). Notably, the correlation between Pixel and IT Security System Adoption is small and statistically insignificant (see Table S5), suggesting that pixel presence does not inherently reflect poor security governance.

Fourth, selection bias in website archiving may impact our analysis, as archived websites in the Wayback Machine are not uniformly available across hospitals. To account for this, we implement Heckman selection models (33–37) where the pixel adoption rate of other hospitals in the same geographic area, excluding the focal hospital, is used to instrument the exclusion restriction variable (2, 38) (Table S8). Additional sensitivity analyses account for potential biases introduced by nonrandom website availability, ensuring the robustness of our findings (Tables S9 and S10).

Further robustness checks include controlling for time invariant factors by removing hospital fixed effects (Table S11), using alternative nonlinear specifications (Table S12), and accounting for the effect of potential hospital consolidations on breach risk (39) by removing hospitals that had a system size change (Table S13).

While our empirical strategy addresses multiple identification threats, we acknowledge that unobserved time-varying confounders may still influence the results. Additionally, we cannot directly observe the causal chain behind each breach. These limitations are inherent to observational research, but the consistency of our findings across robustness checks provides confidence in the core relationship.

## Supplementary Material

pgaf360_Supplementary_Data

## Data Availability

All data used in this study are aggregate or institutional-level and publicly available; no individually identifiable health information was accessed. The final dataset and code for the statistical analysis are available in the following public repository: https://doi.org/10.5281/zenodo.17547225

## Notes

a Due to data limitations associated with the IT Security and Digitization controls, our remaining analyses follow the analyses displayed in the first four columns of Table 1. However, our results remain qualitatively similar in terms of magnitude, sign, and significance even when including these controls in subsequent analyses.

b HIMSS database is not available for researchers after 2018.

c The Python code for pixel detection is available in the following link: https://www.dropbox.com/scl/fo/klzgkdwdp4loon5otrkkm/AKR_EMbwUXuASUrOQiT_hvg?rlkey=8nw0glzkn3m85alqyxrvylbf4&st=ui37hes9&dl=0
