# Supplementary material for: Beyond the click: Pixel tracking technologies and patient data security in hospitals
Source: PNAS Nexus. 2025 Dec 9;4(12):pgaf360. doi: 10.1093/pnasnexus/pgaf360 (PMC12687351; doi:10.1093/pnasnexus/pgaf360)
Supplement: pgaf360_Supplementary_Data [file pgaf360_supplementary_data.pdf]

Beyond the Click: Pixel Tracking Technologies and Patient Data Security in Hospitals

SUPPLEMENTARY MATERIALS

Additional Statistics

Table S1. Variables

Variable	Description and Source
Pixel-related variables	
Pixel	A dichotomous variable equal to one if a hospital's website has a pixel managed by a third-party domain in year t, and zero otherwise.<br><br>Source: Analysis of hospital websites via Wayback Machine using webXray.
Own Pixel	A dichotomous variable equal to one if a hospital's website has a pixel managed by the hospital or hospital system in year t, and zero otherwise.<br><br>Source: Analysis of hospital websites via Wayback Machine using webXray.
Area Pixel Rate	Percentage of hospitals geographically near hospital *i* at time *t* that have adopted pixels.<br><br>Source: Author calculation.
Dependent variable	
Breach	A dichotomous variable equal to one if a hospital experienced a breach incident in year t, and zero otherwise.<br><br>Source: Privacy Rights Clearinghouse Data Breach Chronology Database. See here: https://privacyrights.org/data-breaches.
Hospital Characteristics	
Assets	A hospital's total assets in millions.<br><br>Source: HCRIS Hospital Provider Cost Report database. See here: https://data.cms.gov/provider-compliance/cost-report/hospital-provider-cost-report.
EHR Adoption	A continuous variable equal to 1 if a hospital adopted all five EHR applications and equal to 0 if it did not any (incrementally increasing with the adoption of each application).<br><br>Source: Healthcare Information and Management Systems Society (HIMSS) database. See here: https://foundation.himss.org/Dorenfest/About.
Faith	A dichotomous variable equal to one if a hospital has a religious affiliation, and zero otherwise.

	Source: The Provider of Services File - Hospital & Non-Hospital Facilities https://data.cms.gov/provider-characteristics/hospitals-and-other-facilities/provider-of-services-file-hospital-non-hospital-facilities
For Profit	A dichotomous variable equal to one if a hospital is a private for-profit hospital, and zero otherwise.<br><br>Source: The Provider of Services File - Hospital & Non-Hospital Facilities
HIE Adoption	A dichotomous variable equal to 1 if a hospital is part of a data exchange network, and 0 otherwise.<br><br>Source: Healthcare Information and Management Systems Society (HIMSS) database.
IT Security System Adoption	A continuous variable equal to 1 if a hospital adopted all four security systems and equal to 0 if it did not adopt any (incrementally increasing with the adoption of each security system).<br><br>Source: Healthcare Information and Management Systems Society (HIMSS) database.
Net Income	A hospital's reported net income in millions.<br><br>Source: HCRIS Hospital Provider Cost Report database.
Number of Beds	Number of beds located at hospital i in year t.<br><br>Source: The Provider of Services File - Hospital & Non-Hospital Facilities
Number of Employees	Number of staff employed by hospital i in year t.<br><br>Source: The Provider of Services File - Hospital & Non-Hospital Facilities
System Size	Number of hospitals with the same system identifier as hospital i in year t within the sample.<br><br>Source: HIMSS database and Agency for Healthcare Research and Quality, Compendium of U.S. Health Systems https://www.ahrq.gov/chsp/data-resources/compendium.html
Teaching	A binary variable equal to one if the number of interns and residents (FTE) is higher than 25% of the hospital bed count, and zero otherwise.<br><br>Source: HCRIS Hospital Provider Cost Report database.

Table S2. Descriptive Statistics

VARIABLE	Obs.	Mean	St. Dev.
Breach	11,013	0.030	0.169
Lag Pixel	10,324	0.600	0.491
Log Number of Employees	11,013	6.318	2.844
Log Number of Beds	11,008	6.032	0.949
System Size	11,013	5.496	10.09
Log Assets	9,037	20.01	1.885
Log Net Income	9,393	9.538	14.53
IT Security System Adoption	3,803	0.817	0.288
EHR Adoption	3,803	0.815	0.112
HIE Adoption	3,803	0.600	0.490
Teaching	11,013	0.216	0.412
Faith	11,013	0.084	0.278
For Profit	11,013	0.103	0.304

Notes: This table presents descriptive statistics for the final sample of hospitals used in our analyses. Refer to Table S1 for variable definitions.

Table S3. Pixel Detection

Year	Hospital Observations	Wayback URL exists	Pixel detected
2012	1,190	681	431
2013	1,193	743	489
2014	1,195	758	488
2015	1,196	800	440
2016	1,196	824	449
2017	1,198	857	488
2018	1,198	862	546
2019	1,199	903	631
2020	1,199	1,034	715
2021	1,200	1,094	781
2022	1,201	1,138	829
2023	1,201	1,201	836
	14,366	10,895	7,123 (65.5%)

Table S4. Breach Types

Year	Breach type					
	# of breach incidents	Unintended Disclosure	Hack	Insider	Other	Unknown
2012	39	0	0	2	14	23
2013	29	4	0	5	7	13
2014	30	4	0	4	11	11
2015	21	6	0	5	3	7
2016	28	3	6	6	3	10
2017	48	7	18	5	7	11
2018	63	10	19	5	11	18
2019	74	18	24	16	1	15
2020	38	2	19	1	5	11
2021	18	2	11	1	0	4
2022	19	1	8	0	3	7
2023	11	0	6	0	0	5
Sum	418	57 (14%)	111 (27%)	50 (12%)	65 (16%)	135 (32%)

Notes: These statistics are based on the full sample including observations with missing URLs.

Table S5. Correlation matrix

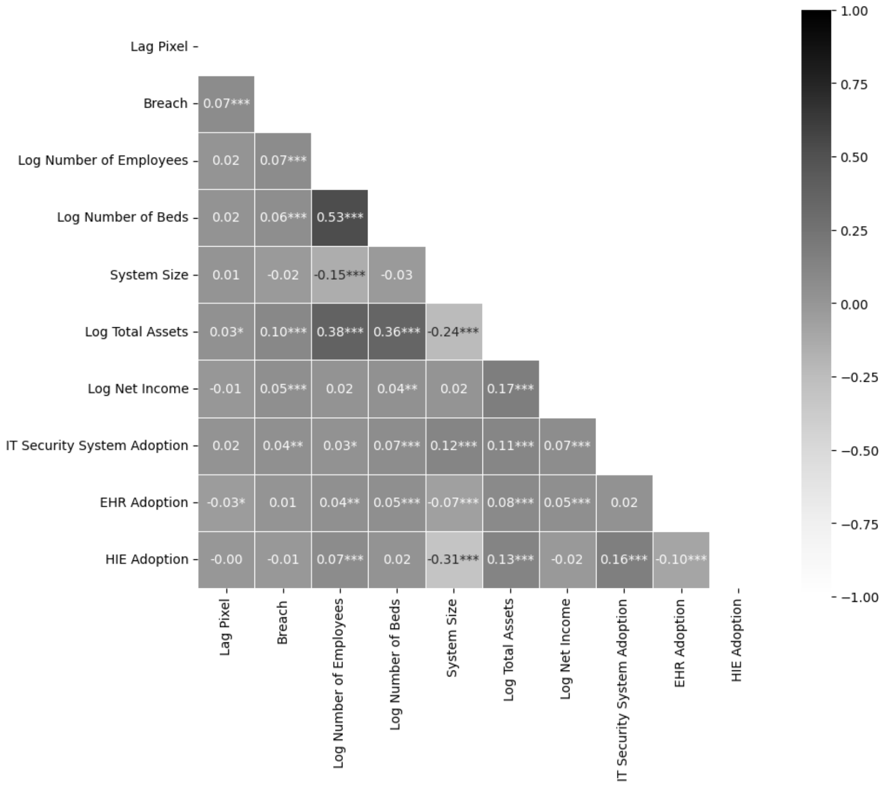

Additional Results

Propensity Score Matching

One of the endogeneity issues in analyzing the relationship between *Pixel* and *Breach* is that hospitals that adopt pixels might be systematically different from hospitals that do not use pixel tracking technology on their websites. We use propensity score matching analysis to address the observable heterogeneity across hospitals. In this analysis, matching covariates are teaching status (*Teaching*), the mission of the hospital (*Faith*), whether the hospital is for-profit (*ForProfit*), *Number of Beds, Net Income, Total Assets,* and the city where the hospital is located. This additional analysis provides consistent results with the main results where *Pixel* is associated with a 2.1% increase in breach probability, which is statistically significant at the 1% significance level.

Hospital-specific time trends

In our main analyses, we account for year fixed-effects for time-specific shocks that are simultaneously experienced by all hospitals. However, different hospitals might be on different time trends in terms of their breach and security issues or affected by shocks in different years. Angrist and Pischke ([1]) recommend using unit-specific time trends to further account for time-variant unobserved factors. To account for within-hospital changes over time, we include a hospital-specific time trend for each hospital, and the results, which are reported in Table S6, remain robust.

Lead and Lag Values of Pixel Use

In our analyses using hospital and year-fixed effects, the main identifying assumption is the parallel trends ([1]). That is, hospitals have similar trends of breach issues before the pixel adoption. To test the timing of the changes in the dependent and independent variables, we analyze the effects of leads and lags of *Pixel* that identify the timing of the effects—that is, whether they precede (lead) or delay (lag) the adoption of pixel tracking technologies.

In Table S7, the current and lead values of *Pixel* are added on top of the lag values. This test follows the idea of a Granger test that analyzes the timing of changes among variables in a time-series context to infer a causal direction[2]. If the breach probability changes due to changes in pixel use and not by other trends or factors across hospitals, we expect to find significant effects of lagged or current *Pixel* on current breach probability. If the future lead values of *Pixel* are significantly correlated with the current breach probability, it would provide evidence of the presence of confounding factors/trends or reverse causality. The results in Table S7 indicate that only the lagged (t-1) values of *Pixel* are significantly correlated with current breaches, supporting the causal direction running from *Pixel* to data breaches.

Selection in Archived Website

The archived websites that are historically identifiable are more likely to have higher user traffic. It is also harder to identify the archived websites in earlier years (Table S3). Overall, the percentage of hospital-year observations with available archived websites is 76%. While the observations without available websites are removed from our main analyses to achieve a more accurate measurement of pixel presence, we conduct a robustness check by assigning no pixel use to the unidentified websites (Table S9). We additionally replace the missing pixel values for the hospital with the pixel values for the focal hospital's systems pixel values (Table S10).

Further, we account for the potential selection into the sample of identifiable archived websites using the Heckman Selection model ([3][4]). In the first step, the availability of archived hospital websites is predicted based on relevant hospital attributes, which are controlled for in Equation 1, along with an exclusion restriction variable. For the exclusion restriction, we use the pixel adoption rate of other hospitals in the same geographic area, excluding the focal hospital. Peer and competition effects are known to be important in influencing healthcare providers'

technology adoption decisions ([5][6]). Thus, other hospitals' pixel adoption in the same area can impact the focal hospital's tendency to adopt pixels.

The first stage generates the inverse Mills ratio for each observation, representing the selection bias due to the availability of archived websites. In the second stage, pixel usage is estimated while controlling for the selection bias captured in the first step ([7]). This two-step process ensures that we account for the non-random missingness of pixel tracking data due to unavailable archived websites. Table S8 presents the results from both the first and second stages of the Heckman analysis. The second stage demonstrates that pixel usage is positively associated with breach risk, even after correcting for the selection bias.

Time invariant characteristics

Time invariant hospital characteristics are known to be determinants of breach risk ([8]). However, these time-invariant characteristics are absorbed by the hospital-fixed effects in our main specification. In an alternative specification, we remove hospital fixed effects to be able to estimate the time-invariant characteristics of the hospitals such as teaching status, mission status, and whether the hospital is for-profit or not. Table S11 presents the results without hospital-fixed effects where time-invariant characteristics are controlled for. This analysis indicates that the results are robust to this alternative specification and teaching hospitals have a significantly higher breach risk. In fact, the results indicate that the coefficient of pixel use is larger when hospital fixed effects are removed, providing evidence that unobserved heterogeneity across hospitals is important to consider for accurate estimates of the impact of pixel use on data breach probability.

Alternative Non-Linear Specifications

Although the linear probability model (LPM) is widely used for binary dependent variables, it has certain limitations. One key issue is that its unconstrained nature can result in

predicted probabilities outside the 0 to 1 range. Additionally, LPM is prone to heteroscedasticity, which can lead to inefficient estimates. While using robust standard errors helps address heteroscedasticity concerns, it is important to verify the robustness of our findings using traditional non-linear models. Therefore, we replicate our analysis using logit and probit models, which naturally constrain the dependent variable to binary outcomes. As shown in Table S12, the results remain consistent across these models.

System Size Change

To account for potential confounding effects due to hospital consolidations, we calculated changes in the system size as a dichotomous variable equal to 1 if hospital i had a change in the *System Size* variable in year t compared to year t-1. We constructed system size using two complementary data sources to cover the full span of our panel. For 2012–2017, we used healthcare system identification from the Healthcare Information and Management Systems Society (HIMSS) database. For 2018–2023, we used information from the Agency for Healthcare Research and Quality's Compendium of U.S. Health Systems. Because the Compendium data are only available from 2016 onward, we used HIMSS data for system identification for earlier years in our sample.

In Table S13, we remove observations within a one-year window around a system size change (the year before, the year of, and the year after), as these periods may carry heightened breach risk ([9]). We conduct an additional robustness check using total system size, including hospitals outside our sample, to capture consolidation events that may not be reflected when measuring system size from our study sample (Table S13, columns 4-6). The results from both tests remain consistent with our main findings, suggesting that the documented relationship between pixel tracking and breach risk is unlikely to be driven by consolidation-related changes in system size.

Table S6. Hospital-specific Time Trends

	Dependent variable: $Breach_{i,t}$			
VARIABLE	(1)	(2)	(3)	(4)
Pixel t-1	0.014***	0.014***	0.014***	0.015***
	(0.003)	(0.003)	(0.003)	(0.004)
Controls	No	Size	System	Financial
Hospital Fixed Effects	Yes	Yes	Yes	Yes
Year Fixed Effects	Yes	Yes	Yes	Yes
Hospital-time trend	Yes	Yes	Yes	Yes
Observations	10,324	10,324	10,324	8,535
R-squared	0.414	0.415	0.417	0.419
Adj. R-squared	0.336	0.337	0.339	0.339

Notes: Refer to Table S1 for variable definitions. Standard errors are reported in parentheses and are clustered by hospital. *** p<0.01, ** p<0.05, * p<0.1.

Table S7. Lead Values of Pixel Use and Data Breaches

	Dependent variable: $Breach_{i,t}$			
VARIABLE	(1)	(2)	(3)	(4)
Pixel t-1	0.014***	0.014***	0.014***	0.015***
	(0.004)	(0.004)	(0.004)	(0.004)
Pixel t	-0.002	-0.002	-0.001	-0.002
	(0.004)	(0.004)	(0.004)	(0.005)
Pixel t+1	-0.000	-0.000	0.000	0.001
	(0.004)	(0.004)	(0.004)	(0.005)
Controls	No	Size	System	Financial
Hospital Fixed Effects	Yes	Yes	Yes	Yes
Year Fixed Effects	Yes	Yes	Yes	Yes
Observations	9,123	9,123	9,123	7,533
R-squared	0.441	0.441	0.443	0.445
Adj. R-squared	0.357	0.358	0.360	0.360

Notes: Refer to Table S1 for variable definitions. Standard errors are reported in parentheses and are clustered by hospital.*** p<0.01, ** p<0.05, * p<0.1.

Table S8. Heckman Selection Model

	(1)	(2)	(3)	(4)
VARIABLE	First Stage	Second Stage	First Stage	Second Stage
Pixel t-1		0.015***		0.016***
		(0.003)		(0.004)
Area Pixel Rate	-0.012***		-0.020***	
	(0.001)		(0.001)	
Controls	System	System	Financials	Financials
Hospital Fixed Effects	Yes	Yes	Yes	Yes
Year Fixed Effects	Yes	Yes	Yes	Yes
Observations	12,204	12,204	12,204	10,016
Wald-Chi	6682	6682	6682	5384

Notes: Refer to Table S1 for variable definitions. Standard errors are reported in parentheses and are clustered by hospital. *** p<0.01, ** p<0.05, * p<0.1.

Table S9. Assign zero to missing values of pixel

VARIABLE	(1)	(2)	(3)	(4)
Pixel t-1	0.010***	0.010***	0.010***	0.010**
	(0.003)	(0.003)	(0.003)	(0.004)
Controls	No	Size	System	Financial
Hospital Fixed Effects	Yes	Yes	Yes	Yes
Year Fixed Effects	Yes	Yes	Yes	Yes
Observations	13,315	13,315	13,315	11,181
R-squared	0.381	0.382	0.382	0.385
Adj. R-squared	0.320	0.320	0.320	0.322

Notes: Refer to Table S1 for variable definitions. Standard errors are reported in parentheses and are clustered by hospital. *** p<0.01, ** p<0.05, * p<0.1.

Table S10. Assign system pixel to missing values of hospital pixel

VARIABLE	(1)	(2)	(3)	(4)
Pixel t-1	0.012***	0.012***	0.013***	0.014***
	(0.003)	(0.003)	(0.003)	(0.004)
Controls	No	Size	System	Financial
Hospital Fixed Effects	Yes	Yes	Yes	Yes
Year Fixed Effects	Yes	Yes	Yes	Yes
Observations	11,344	11,344	11,344	9,377
R-squared	0.410	0.410	0.412	0.413
Adj. R-squared	0.339	0.340	0.341	0.341

Notes: Refer to Table S1 for variable definitions. Standard errors are reported in parentheses and are clustered by hospital. *** p<0.01, ** p<0.05, * p<0.1.

Table S11. Time invariant factors

	Dependent variable: $Breach_{i,t}$			
VARIABLE	(1)	(2)	(3)	(4)
Pixel t-1	0.022***	0.021***	0.021***	0.022***
	(0.005)	(0.005)	(0.005)	(0.005)
Teaching	0.035**	0.027**	0.028**	0.021*
	(0.014)	(0.013)	(0.013)	(0.012)
Faith	0.012	0.008	0.005	0.004
	(0.013)	(0.013)	(0.013)	(0.013)
For Profit	0.005	0.003	-0.006	-0.004
	(0.011)	(0.012)	(0.014)	(0.013)
Controls	No	Size	System	Financial
Hospital Fixed Effects	No	No	No	No
Year Fixed Effects	Yes	Yes	Yes	Yes
Observations	10,324	10,324	10,324	8,535
R-squared	0.048	0.050	0.051	0.051
Adj. R-squared	0.046	0.048	0.049	0.049

Notes: Refer to Table S1 for variable definitions. Standard errors are reported in parentheses and are clustered by hospital. *** p<0.01, ** p<0.05, * p<0.1.

Table S12. Alternative Specifications

	(1)	(2)	(3)	(4)
VARIABLE	Logit	Logit	Probit	Probit
Pixel t-1	0.814***	0.784***	0.446***	0.426***
	(0.229)	(0.231)	(0.127)	(0.128)
Controls	System	Financial	System	Financial
Hospital Fixed Effects	Yes	Yes	Yes	Yes
Year Fixed Effects	Yes	Yes	Yes	Yes
Observations	1,269	1,225	1,269	1,225

Notes: Refer to Table S1 for variable definitions. Standard errors are reported in parentheses and are clustered by hospital. *** p<0.01, ** p<0.05, * p<0.1.

Table S13. Remove observations within one-year window around system size change

VARIABLE	(1) System Size Sample	(2) System Size Sample	(3) System Size Sample	(4) System Size Total	(5) System Size Total	(6) System Size Total
Pixel t-1	0.009**	0.009**	0.009**	0.015**	0.014**	0.014**
	(0.004)	(0.004)	(0.004)	(0.006)	(0.006)	(0.006)
Controls	No	Size	System	No	Size	System
Hospital Fixed Effects	Yes	Yes	Yes	Yes	Yes	Yes
Year Fixed Effects	Yes	Yes	Yes	Yes	Yes	Yes
Observations	5,284	5,284	5,284	4,331	4,331	4,331
R-squared	0.413	0.414	0.443	0.604	0.605	0.605
Adj. R-squared	0.295	0.295	0.330	0.511	0.512	0.512

Notes: Refer to Table S1 for variable definitions. Standard errors are reported in parentheses and are clustered by hospital. *** p<0.01, ** p<0.05, * p<0.1.

Figure S1. A demonstration of manual identification of website URL change for hospital St Vincents East, Birmingham, AL, from 2012 to 2023

<u>*Step 1:*</u> The current hospital website URL is identified as the first research result of Google search St Vincents East, Birmingham, AL. The first Google search result comes back as: https://healthcare.ascension.org/locations/alabama/albir/birmingham-ascension-st-vincents-east

<u>*Step 2:*</u> To identify if there have been URL changes for the hospital website, we look at the number of historical captures on Wayback Machine. In this case, the URL identified in Step 1 was only valid from 2021 to 2023.

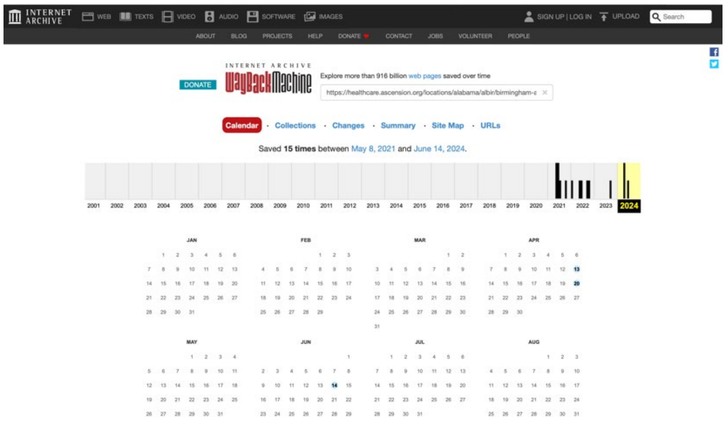

Step3: To identify the hospital URL used before 2021, we first searched hospital system website locations (https://healthcare.ascension.org/Locations) on Wayback Machine to look for locations at Birmingham. The first search result comes back as https://healthcare.ascension.org/Locations/Alabama/ALBIR/Birmingham-St-Vincents-Birmingham

Based on the number of historical captures on Wayback Machine, this URL was valid from 2020 to 2021.

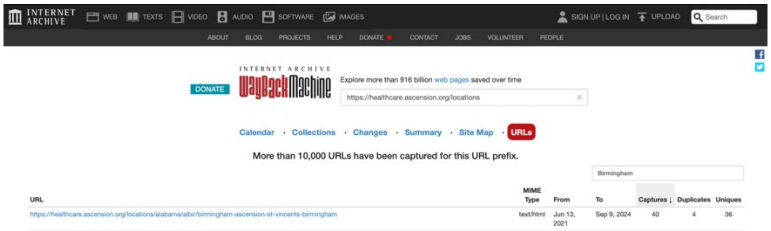

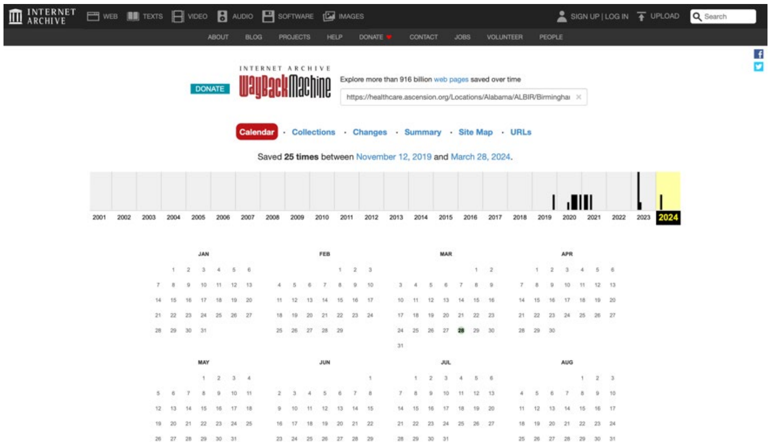

Step4: To identify the historical URL for years before 2019, We searched hospital St Vincents East, Birmingham, AL, on Wayback Machine. The first search result comes back as http://www.stvhs.com/birmingham/. From the number of historical captures on Wayback Machine, this URL was valid from 2012 to 2019.

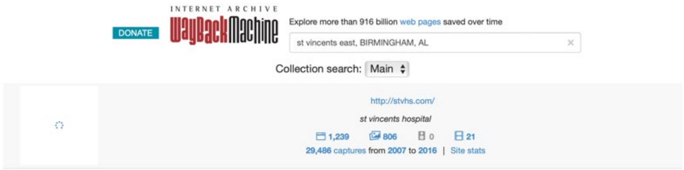

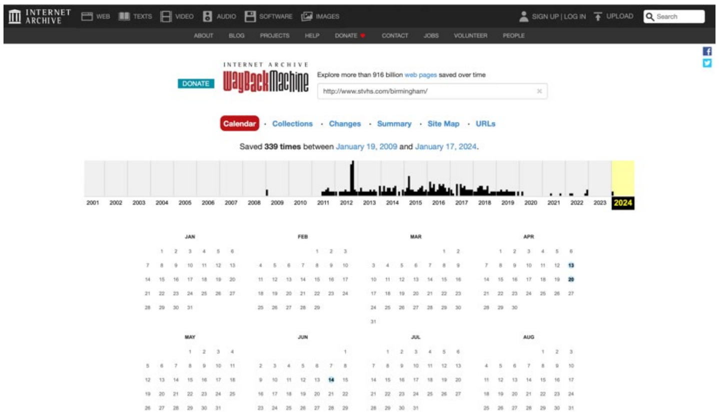

After these manual identification processes, for hospital St Vincents East, Birmingham, AL, the valid website URLs from 2012-2023 are the following:

From 2012-2019, the website URL is:
http://www.stvhs.com/birmingham/

From 2020-2021, the website URL is:
https://healthcare.ascension.org/Locations/Alabama/ALBIR/Birmingham-St-Vincents-Birmingham

From 2022-2023, the website URL is:
https://healthcare.ascension.org/locations/alabama/albir/birmingham-ascension-st-vincents-east

REFERENCES

[1] Angrist, J.D., and J.S. Pischke. "Mostly Harmless Econometrics." *Princeton.edu*, Princeton University Press, 2009, press.princeton.edu/books/paperback/9780691120355/mostly-harmless-econometrics?srsltid=AfmBOopk-IOTzHgF00tmgJ3AJMnJJe-PN6o8CKhh8CNjKgNooe6lImpN.

[2] Friedman, Ari B, et al. "Widespread Third-Party Tracking on Hospital Websites Poses Privacy Risks for Patients and Legal Liability for Hospitals." *Health Affairs*, vol. 42, no. 4, 1 Apr. 2023, pp. 508–515, https://doi.org/10.1377/hlthaff.2022.01205.

[3] Heckman, James J. "The Common Structure of Statistical Models of Truncation, Sample Selection and Limited Dependent Variables and a Simple Estimator for Such Models." *Annals of Economic and Social Measurement*, vol. 5, 1 Oct. 1976, pp. 475–492.

[4] Heckman, James J. “Sample Selection Bias as a Specification Error.” *Econometrica*, vol. 47, no. 1, 1979, pp. 153–161.

[5] Angst, Corey M. “Protect My Privacy or Support the Common-Good? Ethical Questions about Electronic Health Information Exchanges.” *Journal of Business Ethics*, vol. 90, no. S2, Nov. 2009, pp. 169–178, https://doi.org/10.1007/s10551-010-0385-5.

[6] Angst, Corey M., et al. “Social Contagion and Information Technology Diffusion: The Adoption of Electronic Medical Records in U.S. Hospitals.” *Management Science*, vol. 56, no. 8, Aug. 2010, pp. 1219–1241, https://doi.org/10.1287/mnsc.1100.1183.

[7] Wooldridge, Jeffrey M. *Introductory Econometrics: A Modern Approach.* S.L., Cengage Learning, 2009.

[8] Angst, Corey M., et al. “When Do IT Security Investments Matter? Accounting for the Influence of Institutional Factors in the Context of Healthcare Data Breaches.” MIS Quarterly, vol. 41, no. 3, 3 Mar. 2017, pp. 893–916, https://doi.org/10.25300/misq/2017/41.3.10.

[9] Clement, N. (2024). *M&A Effect on Data Breaches in Hospitals: 2010-2022*. Available at: https://weis2023.econinfosec.org/wp-content/uploads/sites/11/2023/06/weis23-clement.pdf
